# Factors associated with health-related quality of life of military policemen in Salvador, Brazil: cross-sectional study

**DOI:** 10.1186/s12955-020-01661-0

**Published:** 2021-01-18

**Authors:** Carla Requião Barreto, Fernando Martins Carvalho, Liliane Lins-Kusterer

**Affiliations:** Postgraduate Program in Health, Environment, and Work, Federal University of Bahia, Largo do Terreiro de Jesus, S/N Centro Histórico, Salvador, Bahia CEP 40026-010 Brazil

**Keywords:** Police, Quality of life, Work capacity evaluation, Cross-sectional studies

## Abstract

**Background:**

Brazil is a violent society and police officers play a fundamental role in this scenario. Police work is a stressful occupation. Dealing with routine violence, police officers must have high standards of physical and mental health. Patrolling the streets involves several risks and stressful situations that may hamper military policemen's quality of life. The identification of factors associated with health-related quality of life may help in planning and providing adequate care to military policemen. This study aimed to identify factors associated with health-related quality of life of military policemen in Salvador, Brazil.

**Methods:**

A cross-sectional design study investigated a random sample of 329 male military police officers, engaged in patrolling the streets of Salvador, Brazil. A structured questionnaire applied to the policemen collected information about age, education, marital status, income, house ownership, car ownership, police rank, working day, alcohol consumption, smoking, frequency of vigorous physical activity, obesity (body mass index ≥ 30.0), and work ability. Health-related quality of life was evaluated through the 36-Item Short Form Health Survey Questionnaire (SF-36). Work ability was assessed through the Work Ability Index questionnaire. Poor work ability was defined by a 7–27 points score. Multiple linear regression models were used to measure the impact of police officers characteristics on the variation in the Physical Component and Mental Component Summary scores.

**Results:**

Normalised scores were below 50.0% for seven out of the eight SF-36 domains and for the two component summaries. The SF-36 Physical Component Summary was significantly (*P* < 0.05) associated with poor work ability, while the Mental Component Summary was associated with poor work ability, excessive alcohol consumption, and younger age (24–34 years). Multivariate analysis estimated that the Physical Component Summary was 7.386 units (%) lower among policemen with poor work ability compared to those with moderate/good/excellent work ability. The Mental Component Summary was 12.755 units lower among those with poor work ability, 5.354 units lower among those with excessive alcohol consumption, and 5.532 units higher among those with younger age.

**Conclusions:**

The military police officers investigated presented low health-related quality of life, associated with younger age, excessive alcohol consumption, and poor work ability.

## Background

During the Vietnam War, between 1955 and 1975, 47,406 American soldiers were killed in action or captivity [1]. In Brazil, in 2018 alone, 57,358 intentional violent deaths were recorded, corresponding to 27.5 deaths per 100,000 inhabitants. Eleven out of 100 deaths were caused by police officers. Conversely, 343 policemen were killed, 256 (75%) of these deaths occurred outside working hours [2].

As other Latin American countries, Brazil is a violent society and police officers play a fundamental role in this scenario. Police work is a stressful occupation. Dealing with routine violence, police officers must have high standards of physical and mental health, which usually deteriorate over time [3]. Patrolling the streets involves dealing with theft, drug-trafficking, criminal behaviour, long working hours, the possibility of being injured or killed, and other stressful situations that may damage their physical [4] and mental health [5].

Information obtained after identification of factors associated with health-related quality of life may be useful to propose adequate policies and to implement effective care to military policemen. This study aimed to identify factors associated with the health-related quality of life (HRQoL) of military policemen in the city of Salvador, in the state of Bahia, Brazil.

## Methods

A cross-sectional study of factors associated with health-related quality of life was carried out in a representative sample of military policemen from Salvador, Bahia, Brazil.

Salvador city had estimated 2.9 million inhabitants in 2016; in 2014, its per capita Domestic Gross Product was R$ 19.5050,84 (equivalent to US$ 8386), ranking 26 among the 5565 Brazilian municipalities [6]. In 2010, the Human Development Index of Salvador was high (HDI = 0.759), ranking 383 among Brazilian municipalities [7]. Salvador is a violent city. In 2014, 1309 cases of homicide were reported in Salvador, corresponding to a rate of 48.1 homicides per 100,000 inhabitantes. In 2014, approximately 11% of the 31,039 military policemen in the State of Bahia worked in Salvador [8].

The accessible population was composed of all the 3500 military police officers from 27 battalions. The study included only males, engaged in visible patrolling in public spaces in the city, and excluded those who were on medical leave, engaged in administrative functions, or in specific situations not related to their core-activity. This study is part of a larger study that evaluated other health aspects in military police officers from Salvador [9].

Between February and April 2014, a trained researcher applied an individual, structured questionnaire to the policemen during their working hours. The policemen were individually interviewed, in a reserved consultation office of the batallion. All randomly selected policemen participated voluntarily in the survey. Information was collected about age (24–34; 35–54 years, using the median as the cut-off point), education (high school; graduate/post graduate), marital status (married; others), income (2–6; > 6 Minimal Wages, equivalent to US$ 1856 in March, 2014, using the mean salary of a soldier as the cut-off point) house ownership (yes; no), car ownership (yes; no), police rank (officer; soldier), working day (≤ 8 h/day; > 8 h/day), excessive alcohol consumption (yes; no—the latter including none and light), smoking (no/ex-smoker; smoker), vigorous physical activity (up to 2 times/week; 3–7 times/week) and obesity(yes; no), defined as body mass index ≥ 30.0 [10].

The policeman’s own perception of their work ability was measured by the Work Ability Index—WAI [11]. The Work Ability Index questionnaire has been translated [12] and validated for the Brazilian population, with satisfactory construct validity and reliability [13] and test–retest reliability [14]. WAI has seven dimensions: current work ability compared to lifetime best (0–10 points), work ability in relation to demands of the job (2–20 points), number of diseases diagnosed by a physician (1–7 points), estimated work impairment due to diseases (1–6 points), sick leave over the past 12 months (1–5 points), own prognosis of work ability 2 years from now (1–7 points), and mental resources (1–4 points). WAI is calculated through the sum of points over the seven dimensions, varying from 7 to 49 points. The score can be classified according to four work ability categories: poor (7–27 points), moderate (28–36 points), good (37–43 points), and excellent (44–49 points) [15]. For the purposes of this study, poor work ability was codified as Yes (WAI 7–27 points) or No (WAI 28–49 points).

Health-related quality of life was evaluated using the 36-Item Short Form Health Survey questionnaire (SF-36) [16], cross-culturally validated to Brazilian Portuguese [17].

A random sample, proportionally stratified by the 27 battalions, was selected. As recommended by the SF-36 manual [18], Cohen's [19] formula was used to calculate a sample size capable of detecting differences of 5.0 points between the sample mean and the mean of a fixed standard. To estimate the population’s standard deviations, the values for males aged 25–34, 35–44, and 44–54 years old were adopted, as demonstrated in Table 10.3 of the SF-36 manual [18]. The value 30.24, related to the Role Physical domain, for men aged 44–54 years, was used, since this was the largest of the three age groups and the eight SF-36 domains. The estimated minimal sample size was n = 289, but this was deliberately inflated by 20%, resulting in a desired sample size of n = 347 policemen. There were no refusals, however 18 individuals returned questionnaires with incomplete answers and were excluded from the study. The final sample size was composed of 329 policemen.

## Data processing and analysis

Based on answers to the SF-36 questionnaire, eight multi-item scales (physical functioning, role limitations due to physical problems, bodily pain, general health perceptions, vitality, social functioning, role limitations due to emotional problems and mental health) were constructed and subsequently aggregated into two Component Summaries: the Physical Component Summary and the Mental Component Summary. The eight scales were scored using raw data (0–100 algorithms), while the respective normalized scores and the scores for the two summary measures was performed using QualityMetric Health Outcomes™ Scoring Software 4.0 [20]. The study was licensed by QualityMetric Health Outcomes™ under number QM025904. The normalization procedure transforms raw scores into a mean of 50% and a standard deviation of 10, taking the United States of America general population as a reference. This transformation achieves the same mean and standard deviation for all SF-36 scales and summary measures. Because of their comparable variance, normalized scores enable comparisons between the respective domain and the component scales. Higher scores represent better health-related quality of life.

The internal consistency of the SF-36 and WAI domains was evaluated using Cronbach's alpha. Values should exceed 0.70, but values ≥ 0.60 are acceptable in exploratory research [21].

Literature discuss whether the WAI questionnaire presents one or two dimensions [22]. In order to access dimensionality, parallel analysis (PA) technique was applied to WAI data, using polychoric correlations and Robust Unweighted Least Squares method for the factor extraction [23].

Bivariate analyses used *t*-tests to compare means from the independent samples. Multivariable linear regression models were used to measure the impact of police officer characteristics on variation in the Physical Component and Mental Component Summary scores. All prediction variables (age, education, marital status, income, house ownership, car ownership, police rank, working day, excessive alcohol consumption, smoking, vigorous physical activity, obesity, and poor work ability) were inserted as a block in each equation, using the default selection method ‘Enter’. Cases presenting studentized residual analysis varying around ± 3.000 standard deviations were identified as outliers. Data were analysed using SPSS version 20.0 (IBM Corp., Armonk, NY, USA).

After explanation of the study objectives, participants signed a free informed consent form. All information related to the participants was confidential. The study was approved by the Ethics Committee in Research on Human Beings of the Medical School of Bahia for opinion number 554,724.

## Results

Normalised scores were below 50.0 for the eight SF-36 domains and the two component summaries, except for vitality (51.8 ± 10.7). Scores for general health (44.6 ± 7.8) and social functioning (43.9 ± 11.1) were particularly low. The internal reliability indices for each domain, measured by Cronbach's alpha coefficient, varied from 0.624 (General Health) to 0.896 (Physical Functioning) (Table 1).

**Table 1:** SF-36 normalized scores (in %) and respective Cronbach's alpha coefficients of 329 military policemen

SF-36	Mean ± SD	Cronbach's alpha
Physical functioning (PF)	49.8 ± 8.6	0.896
Role physical (RP)	46.0 ± 10.7	0.815
Bodily pain (BP)	47.4 ± 11.3	0.863
General health (GH)	44.6 ± 7.8	0.624
Vitality (VT)	51.8 ± 10.7	0.866
Social functioning (SF)	43.9 ± 11.1	0.743
Role emotional (RE)	45.4 ± 12.3	0.828
Mental health (MH)	47.2 ± 12.0	0.856
Physical Component Summary (PCS)	47.9 ± 8.2	–
Mental Component Summary (MCS)	46.4 ± 11.7	–

Parallel analysis suggested only one dimension to represent the model for the WAI data. The reliability of the WAI questionnaire, measured by a Cronbach's alpha = 0.844, was higher than the recommended threshold value (0.70).

The policemen in the study were predominantly married (61.1%), presented low levels of education (64.4%), were soldiers (92.4%), owned a car (70.2%), and worked > 8 h/day (79.6%). They did not practice vigorous physical activity frequently (47.7%), and some were obese (14.3%), smokers (5.8%), heavy drinkers (7.3%), and presented poor work ability (10.3%). Bivariate analyses revealed that the Physical Component Summary was strongly (*P* < 0.05 or less) associated with working day (*P* = 0.023), vigorous physical activity (*P* = 0.045), obesity (*P* = 0.041), and poor work ability (*P* < 0.001). The Mental Component Summary was significantly lower among military policemen from age group 24–34 years (*P* = 0.005), with excessive alcohol consumption (*P* = 0.002) and poor work ability (*P* < 0.001) (Table 2).

**Table 2:** Physical (PCS) and Mental (MCS) Component Summaries scores (%) according to characteristics of military policemen

Characteristic	N	%	PCS			MCS		
			Mean	SD	*P*^a^	Mean	SD	*P*^a^
Education					0.986			0.255
High school	212	64.4	48.0	7.9		46.9	11.4	
Graduated/post grad	117	35.6	47.9	8.8		45.4	12.0	
Marital status					0.822			0.722
Married	201	61.1	47.8	8.2		46.5	12.0	
Others	128	38.9	48.1	8.2		46.1	11.2	
Police rank					0.250			0.832
Officer	25	7.6	49.7	7.2		46.8	12.2	
Soldier	304	92.4	47.8	8.3		46.3	11.6	
Income					0.090			0.231
2–6 min. wages	298	90.6	47.7	8.3		46.1	11.7	
> 6 min. wages	31	9.4	50.3	7.2		48.7	11.6	
Car ownership					0.141			0.071
Yes	231	70.2	48.4	7.8		47.1	11.3	
No	98	29.8	46.8	9.1		44.6	12.3	
House ownership					0.984			0.465
Yes	108	32.8	47.9	9.0		47.0	11.6	
No	221	67.2	47.9	7.9		46.0	11.7	
Working day					0.023			0.431
≤ 8 h/day	67	20.4	50.0	7.6		47.3	10.7	
> 8 h/day	262	79.6	47.4	8.3		46.1	11.9	
Vigorous physical activity					0.045			0.457
Up to 2 times/week	157	47.7	47.0	7.9		45.9	12.0	
3–7 times/week	172	52.3	48.8	8.4		46.8	11.4	
Obese					0.041			0.806
No	282	85.7	48.4	7.8		46.4	11.8	
Yes	47	14.3	45.2	10.1		46.0	11.1	
Smoking					0.152			0.632
No/ex-smoker	310	94.2	48.1	8.2		46.4	11.6	
Smoker	19	5.8	45.3	9.2		45.9	12.4	
Age group					0.177			0.005
24–34 years	150	45.6	48.6	7.8		44.4	12.2	
35–54 years	179	54.4	47.4	8.5		48.0	10.9	
Excessive alcohol consumption					0.069			0.002
No	305	92.7	48.2	8.1		46.9	11.5	
Yes	24	7.3	45.0	9.0		39.4	11.3	
Poor work ability					< 0.001			< 0.001
No	295	89.7	48.8	7.9		47.7	10.7	
Yes	34	10.3	40.6	7.1		34.6	13.0	

^a^Independent samples *t*-test

Multiple linear regression analyses revealed that the Physical Component Summary was associated with poor work ability (*P* < 0.001). Equation 1 estimates that the mean Physical Component Summary was 7.368 units (%) lower among those with poor work ability. Equation 2 revealed that the Mental Component Summary was associated with poor work ability (*P* < 0.001), excessive alcohol consumption (*P* = 0.028), and age (*P* = 0.011). Equation 2 estimates that the mean Mental Component Summary was 3.532 units (%) lower (*P* < 0.006) among those aged 35–54 years, 5.354% lower (*P* < 0.028) among those with excessive alcohol consumption, and 12.755%% lower (*P* < 0.001) among those with poor work ability (Table 3).

**Table 3:** Multiple linear regression equations having PCS and MCS (%) as dependent variable in 329 military policemen

Predictor in the equation (referent)	Equation 1 (Dependent = PCS)				Equation 2 (Dependent = MCS)			
	b^a^	Std error of b	*P*	Beta^b^	b^a^	Std error of b	*P*	Beta^b^
Education (Grad/postgrad.)	− 0.248	0.939	0.792	− 0.014	0.043	2.981	0.974	0.002
Marital status (Married)	− 0.583	0.934	0.533	− 0.035	0.078	1.303	0.952	0.003
Police rank (Officer)	1.189	2.294	0.605	0.038	3.044	3.181	0.339	0.069
Income (> 6 Min. wages)	− 2.515	2.076	0.227	− 0.090	− 3.273	2.880	0.257	− 0.082
Car ownership (Yes)	− 0.776	0.989	0.433	− 0.043	− 1.644	1.372	0.232	− 0.065
Housing ownership (Yes)	0.306	0.943	0.746	0.018	0.308	1.309	0.814	0.012
Working day (≤ 8 h/day)	− 1.139	1.136	0.317	− 0.056	0.190	1.576	0.904	0.007
Vigorous physical activity (3 − 7 times/week)	− 1.327	0.881	0.133	− 0.081	− 0.574	1.222	0.639	− 0.025
Obese (Normal/overweight)	− 1.532	1.299	0.239	− 0.065	0.840	1.802	0.641	0.025
Smoking (No/ex-smoker)	− 1.338	1.912	0.485	− 0.038	0.745	2.652	0.779	0.015
Age group (35–54 years)	− 0.932	0.928	0.316	− 0.057	3.532	1.288	0.006	0.151
Excessive alcohol consumption (No)	− 1.213	1.747	0.488	− 0.039	− 5.354	2.423	0.028	− 0.120
Poor Work Ability (No)	− 7.386	1.484	< 0.001	− 0.275	− 12.755	2.058	< 0.001	− 0.334
Constant	52.733	2.149	< 0.001	–	46.467	4.205	< 0.001	–

^a^Regression coefficient b

^b^Standardized coefficient Beta

Beta (standardized) regression coefficients enable comparisons of the relative effect of each independent variable on the model’s dependent variables. Compared to the other twelve variables in the models, poor work ability presented the highest betas in both Equation 1 (− 0.275) and Equation 2 (− 0.334) (Table 3).

Residual analysis revealed one outlier in Equation 1 (with PCS as the dependent variable), and two outliers in Equation 2 (with MCS as the dependent variable). Excluding these outliers did not substantially change the results in either equation. Collinearity among the predictors did not provide important limitations to the analyses. Tolerance collinearity statistics (1 − R^2^) were always high for both equations, ranging from 0.502 to 0.957. Tolerance statistics close to zero indicates that a variable is almost a linear combination of the other predictors in the model. Adjusted R^2^ was 0.093 in Equation 1 and 0.135 in Equation 2. The Durbin-Watson statistics fell within the acceptable range (1.5–2.5) in Equation 1 (1.972) and in Equation 2 (1.925) (Table 3).

No interaction among variables presented in Table 3 was found, using first-order interaction terms in multiple linear regression analyses. Small numbers in some strata prevented from drawing conclusions about interactions among the predictors investigated.

## Discussion and conclusion

General health and social functioning scores were particularly low for policemen in Salvador. All eight SF-36 scales contributed, with varying weights, to the Physical Component and Mental Component Summary scores. However, physical functioning, role physical, and bodily pain usually contributed more significantly to the Physical Component Summary, whereas social functioning, role emotional, and mental health contributed more significantly to the Mental Component Summary. The vitality, general health, and social functioning scales provided noteworthy contributions to both Physical and Mental Component Summary scores [24].

Reliability was high (> 0.80) for most of the SF-36 domains (PF, RP, BP, VT, RE and MH); good (0.743) for the SF domain, and acceptable (0.642) for the GH domain.

The Physical (*P* < 0.001) and Mental (*P* < 0.001) Component Summaries of the health-related quality of life of the study population presented strong associations with poor work ability. The Work Ability Index is a complex construct that represents interaction between the individual’s resources and their physical, mental and social work demands, the work environment, organizational culture and management. The WAI may be affected by various aspects of a worker's health-related quality of life [15]. Analogously, the SF-36 physical and mental component summaries are also complex constructs that involve a range of aspects related to work ability. Significant relationships were reported between WAI score and all SF-36 dimensions [25, 26].

Compared to the other twelve variables in the models, poor work ability presented the best predictors of the physical and mental component summaries, by some distance. A value 7.386 units (%) lower was estimated for mean PCS among policemen with poor work ability, compared with policemen with moderate/good/excellent work ability. For mean MCS, the estimated value was 12.755% lower among policemen with poor work ability.

Minimal Clinically Important Difference (MCID) can be defined as the smallest difference in score in the domain of interest which is perceived to be beneficial or harmful, and that would imply in a change in patient´s management. Ideally, the MCID should be ascertained to each particular study population [27]. The MCID for group-level is necessarily smaller than for individual patient-level, because of greater measurement error inherent to patient's quality of life scores [28]. To the best of our knowledge, the MCID for the SF-36 Component Summaries (PCS and MCS) have not yet been determined for general or occupational populations, like military policemen.

It is unacceptable for 10.3% of these military policemen to present poor work ability. Policemen should be physically fit in order to undertake patrols. Difficulty in patrolling the street may be harder to policemen with poor work ability, since they also present lower mental health component of their health-related quality of life.

Policemen who were heavy drinkers presented MCS scores 5.354 lower than those who did not drink alcohol or were light drinkers. The inherently stressful work in the police service may be an important contributor to alcohol use. Excessive alcohol consumption is a frequent problem among a number of occupational groups in the USA, such as miners (17.5%) and construction workers (16.5%), aged between 18 and 64 [29]. In a large survey among male police officers from California and New York City, 7.8% met criteria that indicated probable lifetime alcohol abuse or dependence [30]. This figure is similar to that found for the self-reported excessive alcohol consumption in our study: 7.3%.

Multiple regression analysis revealed a positive, statistically significant (b = 0.237; *P* = 0.006) association between age and the mental component of health-related quality of life (MCS). This association probably reflects a healthy worker survivor effect [31]. Stressful working conditions can affect policemen’s mental health status over time, leading them to choose to leave the workforce, while those presenting optimal physical and mental health tend to remain in the job.

Some limitations of this study need to be addressed. Its cross-sectional design precludes the possibility of establishing causality among our key variables, mainly because of a lack of knowledge about their temporal sequence. Bi-directional cause-effect relationships between HRQoL component summaries (PCS and MCS) and poor work ability cannot be ruled out. Low values of adjusted R^2^ (0.093 for PCS and 0.135 for MCS) can be due to: (1) relevant predictors were not included in the model; and/or (2) a great proportion of the relationship among the outcomes and the predictor variables investigated was not linear.

Despite these limitations, this pioneering study was the first to evaluate the HRQoL of military policemen in a representative sample from a large city in Brazil. The physical and mental components of HRQoL were strongly associated with poor work ability. The mental component of HRQoL was lower among heavy drinkers and those who were younger. Given the key role military policemen play in community safety, these results are worrisome and deserve the attention of military police corporate managers in order to take preventive measures to protect these workers’ health. Indeed, the results of this study provided valuable information to the military corporation health department to plan and establish its first Medical Control Program [32].

In conclusion, military police officers from Salvador city presented low health-related quality of life, associated with excessive alcohol consumption and poor work ability, which may hamper their professional activities. The association between younger age and lower mental component of their health-related quality of life (MCS) is probably due to a healthy worker survivor effect. These findings were important in planning the activities of a health care program for this particularly vulnerable occupational group.

## Data Availability

The data supporting the findings of this study are available from Liliane Lins-Kusterer but restrictions apply to the availabilityof these data, which were used under license for the current study, and so are not publicly available. Data are however available from the authors upon reasonable request and with permission of Liliane Lins-Kusterer.

## Abbreviations

SF-36: 36-Item Short Form Health Survey Questionnaire
PF: Physical functioning
RP: Role physical
BP: Bodily pain
GH: General health
VT: Vitaliy
SF: Social functioning
RE: Role emotional
MH: Mental health
PCS: Physical Component Summary
MCS: Mental Component Summary
sd: Standard deviation
WAI: Work Ability Index
MCID: Minimal Clinically Important Difference
